# Comprehensive comparison of dual-energy computed tomography and magnetic resonance imaging for the assessment of bone marrow edema and fracture lines in acute vertebral fractures

**DOI:** 10.1007/s00330-021-08081-8

**Published:** 2021-07-02

**Authors:** Marco Cavallaro, Tommaso D’Angelo, Moritz H. Albrecht, Ibrahim Yel, Simon S. Martin, Julian L. Wichmann, Lukas Lenga, Silvio Mazziotti, Alfredo Blandino, Giorgio Ascenti, Marcello Longo, Thomas J. Vogl, Christian Booz

**Affiliations:** 1 Division of Experimental Imaging, Department of Diagnostic and Interventional Radiology, University Hospital Frankfurt, Theodor-Stern-Kai 7, 60590 Frankfurt am Main, Germany; 2 Department of Biomedical Sciences and Morphological and Functional Imaging, University of Messina, Via Consolare Valeria 1, 98100 Messina, Italy; 3 Department of Diagnostic and Interventional Radiology, University Hospital Frankfurt, Theodor-Stern-Kai 7, 60590 Frankfurt am Main, Germany

**Keywords:** Radiology, Multidetector computed tomography, Magnetic resonance imaging, Spinal fractures, Retrospective study

## Abstract

**Objectives:**

To compare dual-energy CT (DECT) and MRI for assessing presence and extent of traumatic bone marrow edema (BME) and fracture line depiction in acute vertebral fractures.

**Methods:**

Eighty-eight consecutive patients who underwent dual-source DECT and 3-T MRI of the spine were retrospectively analyzed. Five radiologists assessed all vertebrae for presence and extent of BME and for identification of acute fracture lines on MRI and, after 12 weeks, on DECT series. Additionally, image quality, image noise, and diagnostic confidence for overall diagnosis of acute vertebral fracture were assessed. Quantitative analysis of CT numbers was performed by a sixth radiologist. Two radiologists analyzed MRI and grayscale DECT series to define the reference standard.

**Results:**

For assessing BME presence and extent, DECT showed high sensitivity (89% and 84%, respectively) and specificity (98% in both), and similarly high diagnostic confidence compared to MRI (2.30 vs. 2.32; range 0–3) for the detection of BME (*p* = .72). For evaluating acute fracture lines, MRI achieved high specificity (95%), moderate sensitivity (76%), and a significantly lower diagnostic confidence compared to DECT (2.42 vs. 2.62, range 0–3) (*p* < .001). A cutoff value of − 0.43 HU provided a sensitivity of 89% and a specificity of 90% for diagnosing BME, with an overall AUC of 0.96.

**Conclusions:**

DECT and MRI provide high diagnostic confidence and image quality for assessing acute vertebral fractures. While DECT achieved high overall diagnostic accuracy in the analysis of BME presence and extent, MRI provided moderate sensitivity and lower confidence for evaluating fracture lines.

**Key Points:**

• *In the setting of spinal trauma, dual-energy CT (DECT) is highly accurate in the evaluation of acute vertebral fractures and bone marrow edema presence and extent.*

• *MRI provides moderate sensitivity and lower diagnostic confidence for the depiction of acute fracture lines, when compared to DECT, which might result in potentially inaccurate and underestimated severity assessment of injuries in certain cases when no fracture lines are visible on MRI.*

• *DECT may represent a valid imaging alternative to MRI in specific settings of acute spinal trauma and in follow-up examinations, especially in elderly or unstable patients and in cases of subtle or complex orientated fracture lines.*

## Introduction

In the setting of spinal trauma, radiologic diagnosis of acute vertebral fractures based on exclusive morphologic signs of fracture lines can be challenging, especially in elderly patients presenting with older osteoporotic fractures [1]. In this context, detection of bone marrow edema (BME) as a sign of acute injury can substantially facilitate a more accurate diagnosis [2].

Magnetic resonance imaging (MRI) adequately shows BME and represents the current imaging gold standard for diagnosis of acute vertebral fractures [3, 4]. However, MRI suffers from limitations, such as reduced depiction of osseous structures and long examination times [1, 5, 6].

Computed tomography (CT) represents the gold standard for morphologic assessment of bony structures and fracture lines due to high spatial resolution, enabling short examination times [7]. However, the depiction of BME in conventional CT is impaired by trabecular bone overlying bone marrow [8]. As a technical development, dual-energy CT (DECT) is able to overcome this limitation by distinguishing certain materials based on distinct attenuation profiles through application of two different x-ray energy spectra. DECT usage has significantly increased in clinical routine and numerous post-processing algorithms have been developed [9–11]. In this context, DECT-derived virtual non-calcium (VNCa) images allow for subtraction of calcium signal from trabecular bone, enabling visualization of BME [12, 13]. Regarding acute vertebral fractures, several studies demonstrated high diagnostic accuracy of VNCa series exclusively for the assessment of BME compared with MRI [1, 6, 14].

DECT may be considered as a comprehensive technique providing both detailed information on 3D orientation of fracture lines and BME. However, a comprehensive combined analysis of DECT and MRI regarding both types of information has not been performed to date, which may be of special clinical relevance in the perspective of an accurate assessment of spinal trauma severity and corresponding timely start of appropriate therapy. Therefore, the aim of our study was to thoroughly compare DECT and MRI in the clinical setting of acute vertebral fractures, with regard to diagnostic accuracy, diagnostic confidence, and image quality for the assessment of fracture lines and BME.

## Materials and methods

This retrospective study was approved by the local institutional review board (IRB).

### Patient selection

We enrolled 163 consecutive patients with acute spinal trauma who had undergone both clinically indicated DECT and MRI of the thoracic and/or lumbar spine between December 2015 and July 2019. To minimize potential time-related bias and to ensure comparability, 31 patients with an examination interval of more than one week and 12 patients with inadequate DECT or MRI image quality (severe motion or streaking artifacts) were excluded. Furthermore, patients with a known malignancy of the spine (n = 21) and spondylodiscitis (n = 11) were not included as well, resulting in a final study group consisting of 88 patients. In these patients, eleven vertebral bodies were excluded from analysis due to the presence of dorsal metal implants/inter-body spacers (n = 4), inserted cement after vertebroplasty/kyphoplasty (n = 3), or subtotal vertebral collapse (n = 4), with a final number of 730 assessed vertebral bodies.

### DECT protocol

All CT examinations were performed using a 192-slice third-generation dual-source DECT scanner (Somatom Force; Siemens Healthineers). Tube settings were as follows: tube A, 90 kVp, 220 mAs, and tube B, Sn150 kVp, 138 mAs. Acquisition settings were as follows: collimation, 128 × 0.6 mm; pitch, 0.6; and rotation time, 500 ms. No intravenous contrast material was used. A real-time automated attenuation-based tube current modulation software was implemented (Care Dose 4D; Siemens). The mean volume CT dose index was 9.9 mGy ± 2.9 (range, 5.2–17.4 mGy), and the mean dose-length product was 332.2 mGy · cm ± 150.8 (range, 115.1–720.6 mGy · cm). Acquisition times of each DECT scan were noted and averaged.

Three different image sets were acquired in each DECT examination: 90 kVp, Sn150 kVp, and standard linearly blended M_0.5 images to resemble a standard single-energy 120-kVp image. For fracture detection, axial, coronal, and sagittal reconstructions (section thickness, 1 mm; increment, 0.75 mm) were reconstructed with a bone kernel (Br69f). For dual-energy post-processing, reconstructions in the axial, coronal, and sagittal planes (section thickness, 1 mm; increment, 0.75 mm) were created with a medium-soft convolution kernel (Qr40 with an advanced model-based iterative reconstruction [ADMIRE] strength level of 3). Post-processing was performed with a three-dimensional workstation (*bone marrow* algorithm, syngo.via version VB30B, Siemens Healthineers) by using a three-material decomposition algorithm, which differentiates bone mineral and red and yellow bone marrow [13, 15]. The resulting VNCa images were displayed as color-coded maps, modifying the following parameters from the vendor pre-set dual-energy bone marrow settings on the basis of empirical clinical experience: resolution, 3; maximum attenuation value, 650 Hounsfield units (HU); and bone threshold, 115 HU. Colored VNCa images and standard grayscale DECT series were sent to the picture archiving and communication system (PACS).

### MRI protocol

MRI examinations were performed on a 3.0-T system (Magnetom PrismaFit; Siemens Healthineers) without contrast administration. The scan protocol consisted of sagittal T1-weighted spin-echo (matrix size, 288 × 384; section thickness, 4 mm; repetition time, 650 ms; echo time, 10 ms), axial and sagittal T2-weighted fast spin-echo (matrix size, 358 × 448; section thickness, 4 mm; repetition time, 4000 ms; echo time, 89 ms), and sagittal turbo inversion recovery magnitude (TIRM) (matrix size, 388 × 384; section thickness, 4 mm; repetition time, 3500 ms; echo time, 39 ms) sequences. Acquisition times of each MRI examination were noted and averaged.

### Subjective image analysis

Images were evaluated with a dedicated PACS workstation (Centricity 4.2; GE Healthcare). To set the reference standard, two experienced board-certified radiologists (T.J.V. and J.L.W. with 33 and 8 years of musculoskeletal [MSK] imaging experience, respectively) assessed, in consensus reading sessions, the presence of traumatic BME and acute fracture lines analyzing MR and standard grayscale CT image series respectively, using a 4-point score (0 = no; 1 = probably no; 2 = likely; 3 = distinct presence). Osteoporotic chronic fractured vertebrae were scored 0 as well. Readers also assessed BME extent evaluating MR sagittal TIRM series, and each vertebral body was divided into four anatomic segments (upper-anterior, upper-posterior, lower-anterior, and lower-posterior segments), with each segment representing 25% of the vertebral volume. Depending on the number of segments in which BME extended, a score between 1 (edema confined within one quarter of the vertebral body) and 4 (edema involving all four segments) was assigned.

After the reference standard had been defined, all evaluations were performed by five independent radiologists (M.H.A., L.L., and S.S.M.: board-certified radiologists with 5 years of MSK imaging experience; I.Y. and C.B.: radiology residents with 4 years of MSK imaging experience), who analyzed all MR image datasets in random order. All observers were blinded to reference standard information. Readers initially assessed BME presence on a per-vertebra level (scores 0–3: from no to distinct presence) and BME extent on a per-segment level (scores 1–4: from ¼ to all vertebral body), employing the same 4-point-based scoring systems and the same division of vertebral bodies used in the reference standard session. Subsequently, each vertebral body considered positive for BME was evaluated in all MRI sequences for presence of acute fracture lines. Finally, image quality, image noise, and diagnostic confidence for overall diagnosis of acute vertebral fracture on MRI series were assessed by using 5-point Likert scales (ranging from 1 = unacceptable to 5 = excellent).

After a 12-week interval, readers independently evaluated all DECT examinations, presented in random order. First, readers analyzed VNCa series for presence and extent of traumatic BME by using the same classification systems as previously described. In order to avoid potential artifacts, only BME that was more than 2 mm in distance from adjacent cortical bone was considered for analysis [13, 16]. Subsequently, readers assessed each edematous vertebral body for presence of acute traumatic fracture lines on standard grayscale DECT images reconstructed with bone kernel. The same 4-grade classification for diagnostic confidence used for MRI was adopted. Eventually, image quality, image noise, and confidence for overall diagnosis of acute vertebral fracture were evaluated in all CT series by using the previously described five-point Likert scales. Readers could modify window settings for MRI and CT analysis according to their preference.

### Objective image analysis

Objective image analysis of colored VNCa series was assessed by a sixth reader (M.C., board-certified radiologist with 5 years of MSK imaging experience). CT numbers were obtained from sagittal VNCa images by placing circular regions of interest (ROIs) in each vertebral body and in subcutaneous fat. In every vertebra, ROIs were placed in areas with the highest signal intensity and were carefully drawn with a distance of more than 2 mm from adjacent cortical bone. For all vertebrae without apparent BME, circular ROIs of 100 mm^2^ were placed randomly in the center of the vertebral body. Attenuation values (in HU) and 1-fold standard deviation (SD) from three repeated measurements were recorded and averaged. Signal-to-noise ratio (SNR) values were calculated for each vertebral body using the following equation: (HU vertebral body/SD fat).

### Statistical analysis

Statistical analysis was performed by application of commercially available software (SPSS Statistics, version 26.0). The Shapiro-Wilk test and histograms were used to evaluate the normality of data distribution. Normally distributed data were expressed as means ± standard deviation and analyzed using t tests, while non-normal distributions were shown as medians and interquartile ranges (IQR) and compared with Wilcoxon signed-rank test for paired data. Inter-reader agreement was evaluated by means of weighted Fleiss’ κ according to Landis and Koch [17]. For all statistical tests, a *p* value less than 0.05 was considered significant.

We performed a vertebra-based analysis, computing sensitivity, specificity, positive predictive and negative predictive values (PPV and NPV), and accuracy values with 95% confidence intervals (CI) on VNCa series for the presence of BME and on MR images for the depiction of acute traumatic fracture lines. MRI and standard grayscale DECT series served as the reference standard, respectively. In this context, ratings of 0 and 1 indicated negative results (absence of BME and acute fracture lines), while scores of 2 and 3 indicated positive results (presence of BME and acute fracture lines). An analogous analysis with the calculation of the same values was conducted on VNCa images for the evaluation of BME extent in vertebral bodies on a per-segment basis, with MRI representing the reference standard.

The receiver operating characteristic (ROC) curve analysis and calculation of the area under the curve (AUC) were employed to evaluate CT attenuation values of every vertebra derived from VNCa images, with MRI serving as standard of reference, to ascertain the cutoff CT numbers so as to provide the highest accuracy for the differentiation of BME. From these cutoff values, sensitivity and specificity values were computed.

## Results

**Fig. 1:**
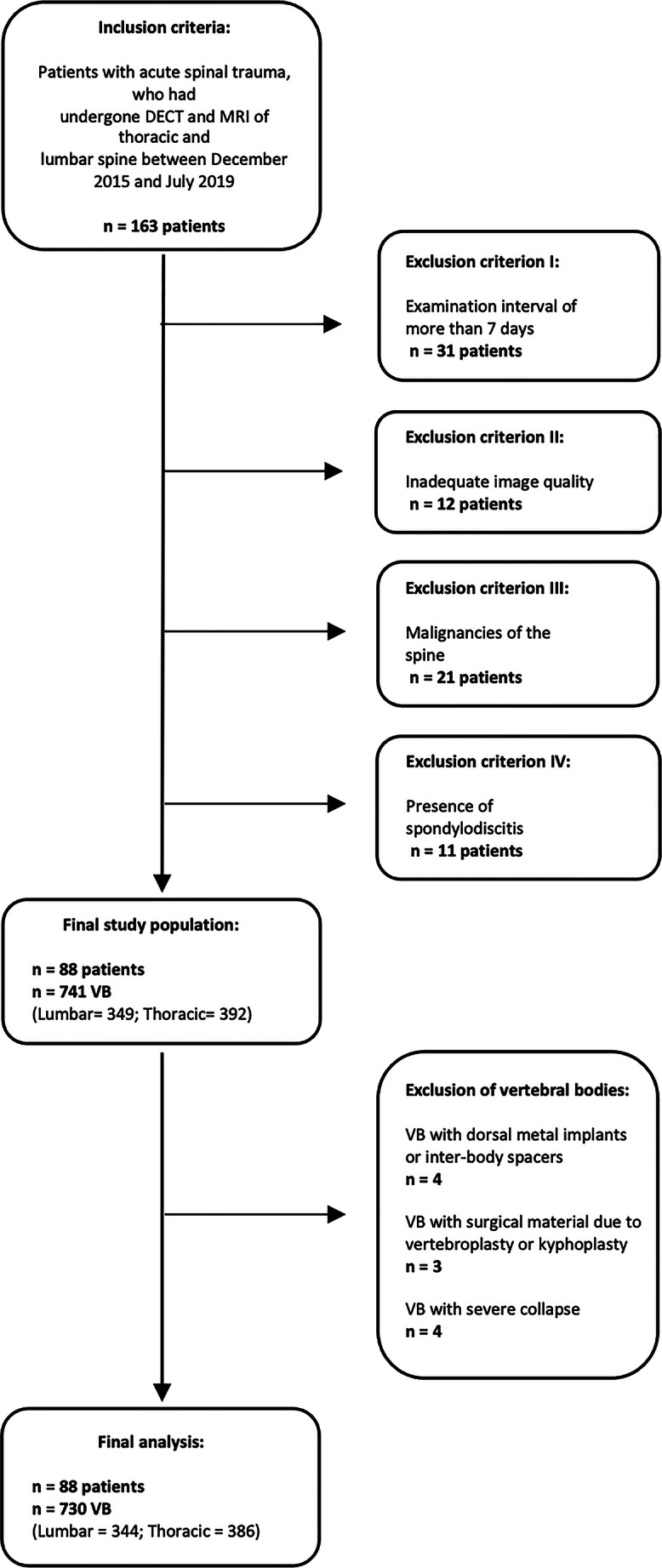
Flow chart of patient inclusion. *VB* vertebral bodies

A total of 730 vertebral bodies (lumbar, n = 344; thoracic, n = 386) in 88 patients (38 males; mean age, 68 ± 16 years) were assessed (Fig. 1, Table 1). MRI revealed 116 edematous vertebrae and 442 segments with signs of BME. Grayscale DECT series showed 89 vertebrae with acute traumatic fracture lines; all of them were among the 116 vertebrae with BME as indicated on MRI. Median interval between DECT and MRI was 2 days (range, 0–7 days). Mean acquisition times were significantly lower for DECT compared to MRI (*p* <.001). Specifically, mean acquisition times were 21 s (range, 18–32 s) and 1103 s (range, 902–1306 s) for DECT and MRI, respectively.

**Table 1:** Patient characteristics

Characteristic	Value
Number of patients	88
Mean age ± SD, range	68 ± 16, 24–91
Mean BMI ± SD, range	24 ± 3, 16–35
Women	50/88 (57)*
Mean age of women ± SD, range	70 ± 16, 24–90
Mean BMI of women ± SD, range	28 ± 3, 16–35
Men	38/88 (43)*
Mean age of men ± SD, range	65 ± 15, 37–91
Mean BMI of men ± SD, range	21 ± 4, 18–34

Age values are expressed in years

*SD* standard deviation, *BMI* body mass index (kg/m^2^)

*Data are numerators and denominators, with percentages in parentheses

### Subjective image analysis

#### Bone marrow edema

**Fig. 2:**
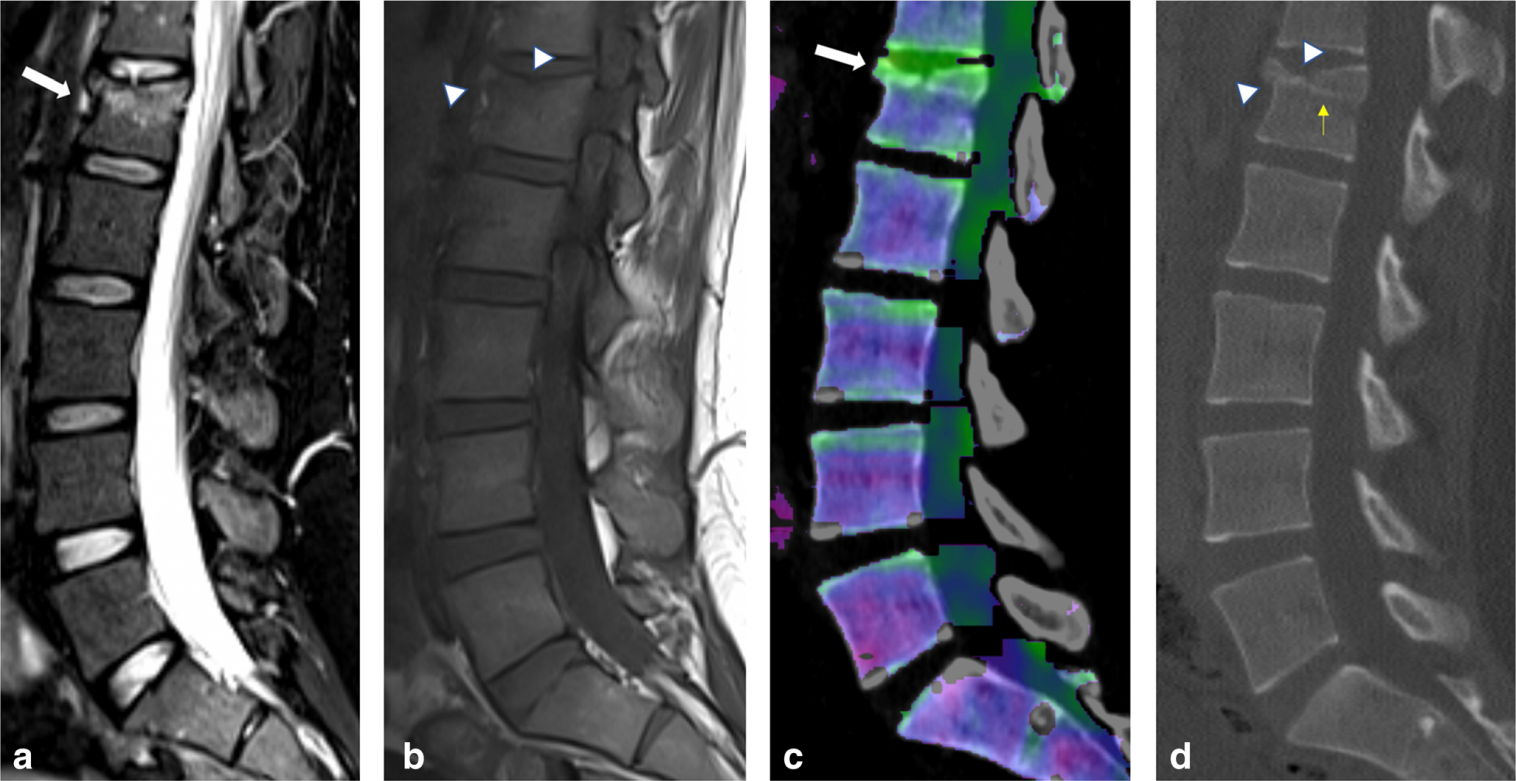
Twenty-six-year-old woman presenting with spinal trauma after motorcycle accident. **a** Sagittal turbo inversion recovery magnitude (TIRM)–magnetic resonance imaging (MRI) series showing bone marrow edema (BME) in the upper two quadrants of L1 (*arrow*). **b** Sagittal spin-echo (SE) T1-weighted MRI demonstrating two distinct acute fracture lines affecting the anterior and upper (*arrowheads*) cortical surfaces. **c** Dual-energy computed tomography (DECT)–virtual non-calcium (VNCa) images depicting BME in the two upper quadrants (*arrow*, displayed as green area). There was a complete agreement between DECT and MRI concerning presence, extent (2 quadrants) and diagnostic confidence (score, 3) regarding BME detection. **d** Sagittal grayscale DECT series not only showing the two cortical fracture lines (*arrowheads*), but also a horizontal hyperdense line indicating trabecular impaction (*yellow arrow*), detected and interpreted as posterior edge involvement by all readers, which was missed on sagittal T1-weighted MRI series by each reader. Confidence in depicting fracture lines was rated as high (score 3) by 5/5 readers on both MRI and DECT series

VNCa reconstructions yielded excellent overall sensitivity (517/580 [89%]), specificity (3012/3070 [98%]), PPV (517/575 [90%]), NPV (3012/3075 [98%]), and accuracy (3529/3650 [97%]) for the assessment of BME per vertebra, as well as optimal overall sensitivity (1847/2210 [84%]), specificity (12,135/12,390 [98%]), PPV (1847/2102 [88%]), NPV (12,135/12,498 [97%]), and accuracy (13,982/14,600 [96%]) for the assessment of BME extent within vertebral bodies (Tables 2 and 3). There was high diagnostic confidence regarding BME presence both on VNCa (average score, 2.30) and MR images (average score, 2.32), without significant differences (*p* = 0.72) (Table 4). Inter-reader agreement was substantial for MRI (κ = 0.63) as well as for VNCa (κ = 0.66) (*p* < .001) in assessing the presence of BME. When evaluating BME extent, readers reported a mean score of 2.78 for MRI and of 2.79 for VNCa reconstructions without significant differences (*p* = 0.57). Inter-reader agreement was good for both MRI (κ = 0.67) and VNCa (κ = 0.66) (*p* = 0.34). Figure 2 shows an example case of high correlation for BME depiction between MRI and VNCa.

**Table 2:** Vertebra-based diagnostic accuracy results of every reader using color-coded virtual non-calcium (VNCa) images for the assessment of bone marrow edema (BME) presence in vertebral bodies

	Sensitivity	Specificity	PPV	NPV	Accuracy
Overall	517/580 (0.89) [0.86–0.92]	3012/3070 (0.98) [0.98–0.99]	517/575 (0.90) [0.87–0.92]	3012/3075 (0.98) [0.97–0.98]	3529/3650 (0.97) [0.96–0.97]
Reader 1	107/116 (0.92) [0.86–0.96]	604/614 (0.98) [0.97–99]	107/117 (0.91) [0.85–0.95]	604/613 (0.98) [0.97–0.98]	711/730 (0.97) [0.96–0.98]
Reader 2	108/116 (0.93) [0.87–0.97]	602/614 (0.98) [0.97–0.99]	108/120 (0.90) [0.84–0.94]	602/610 (0.99) [0.97–0.99]	710/730 (0.97) [0.96–0.98]
Reader 3	103/116 (0.89) [0.82–0.94]	599/614 (0.98) [0.96–0.99]	103/118 (0.87) [0.81–0.92]	599/612 (0.98) [0.97–0.99]	702/730 (0.96) [0.95–0.97]
Reader 4	99/116 (0.85) [0.78–0.91]	603/614 (0.98) [0.97–0.99]	99/110 (0.90) [0.83–0.94]	603/620 (0.97) [0.96–0.98]	702/730 (0.96) [0.95–0.97]
Reader 5	100/116 (0.86) [0.79–0.92]	604/614 (0.98) [0.97–0.99]	100/110 (0.91) [0.84–0.95]	604/620 (0.97) [0.96–0.98]	704/730 (0.96) [0.95–0.98]

Statistical measures are presented as fractions and decimal values (round brackets), with 95% confidence intervals in square brackets.

*PPV* positive predictive value, *NPV* negative predictive value

**Table 3:** Segment-based diagnostic accuracy results of each reader using color-coded virtual non-calcium (VNCa) images for the assessment of bone marrow edema (BME) extent in vertebral bodies

	Sensitivity	Specificity	PPV	NPV	Accuracy
Overall	1847/2210 (0.84) [0.82–0.85]	12135/12390 (0.98) [0.98–0.98]	1847/2102 (0.88) [0.87–0.89]	12135/12498 (0.97) [0.97–0.97]	13982/14600 (0.96) [0.95–0.96]
Reader 1	409/442 (0.93) [0.90–0.95]	2454/2478 (0.99) [0.98–0.99]	409/433 (0.94) [0.92–0.96]	2454/2487 (0.99) [0.98–0.99]	2863/2920 (0.98) [0.97–0.99]
Reader 2	384/442 (0.87) [0.83–0.90]	2417/2478 (0.98) [0.97–0.98]	384/445 (0.86) [0.83–0.89]	2417/2475 (0.98) [0.97–0.98]	2801/2920 (0.96) [0.95–0.97]
Reader 3	353/442 (0.80) [0.76–0.84]	2421/2478 (0.98) [0.97–0.98]	353/410 (0.86) [0.83–0.89]	2421/2510 (0.96) [0.96–0.97]	2774/2920 (0.95) [0.94–0.96]
Reader 4	352/442 (0.80) [0.76–0.83]	2424/2478 (0.98) [0.97–0.98]	352/406 (0.87) [0.83–0.90]	2424/2514 (0.96) [0.96–0.97]	2776/2920 (0.95) [0.94–0.96]
Reader 5	349/442 (0.79) [0.75–0.83]	2419/2478 (0.98) [0.97–0.98]	349/408 (0.86) [0.82–0.88]	2419/2512 (0.96) [0.96–0.97]	2768/2920 (0.95) [0.94–0.96]

Statistical measures are presented as fractions and decimal values (round brackets), with 95% confidence intervals in square brackets

*PPV* positive predictive value, *NPV* negative predictive value

**Table 4:** Readers’ confidence scores for assessing bone marrow edema (BME) and acute vertebral fracture lines using magnetic resonance imaging (MRI) and dual-energy computed tomography (DECT)

	BME		Fracture lines	
	DECT VNCa	MRI	Grayscale DECT	MRI
Average	2.30 ± 0.87	2.32 ± 0.87	2.62 ± 0.65	2.42 ± 0.69
Reader 1	2.43 ± 0.83	2.44 ± 0.85	2.68 ± 0.66	2.52 ± 0.69
Reader 2	2.38 ± 0.86	2.36 ± 0.89	2.71 ± 0.60	2.55 ± 0.66
Reader 3	2.71 ± 0.63	2.62 ± 0.74	2.77 ± 0.55	2.62 ± 0.62
Reader 4	2.67 ± 0.66	2.73 ± 0.65	2.72 ± 0.59	2.47 ± 0.68
Reader 5	2.68 ± 0.67	2.72 ± 0.63	2.69 ± 0.63	2.39 ± 0.76

*VNCa* virtual non-calcium

Average and individual readers’ confidence score for identification of BME (BME presence score 1–3) and acute traumatic fracture lines (fracture line detection score 1–3). Data are given as mean ± standard deviation

#### Fracture lines

**Fig. 3:**
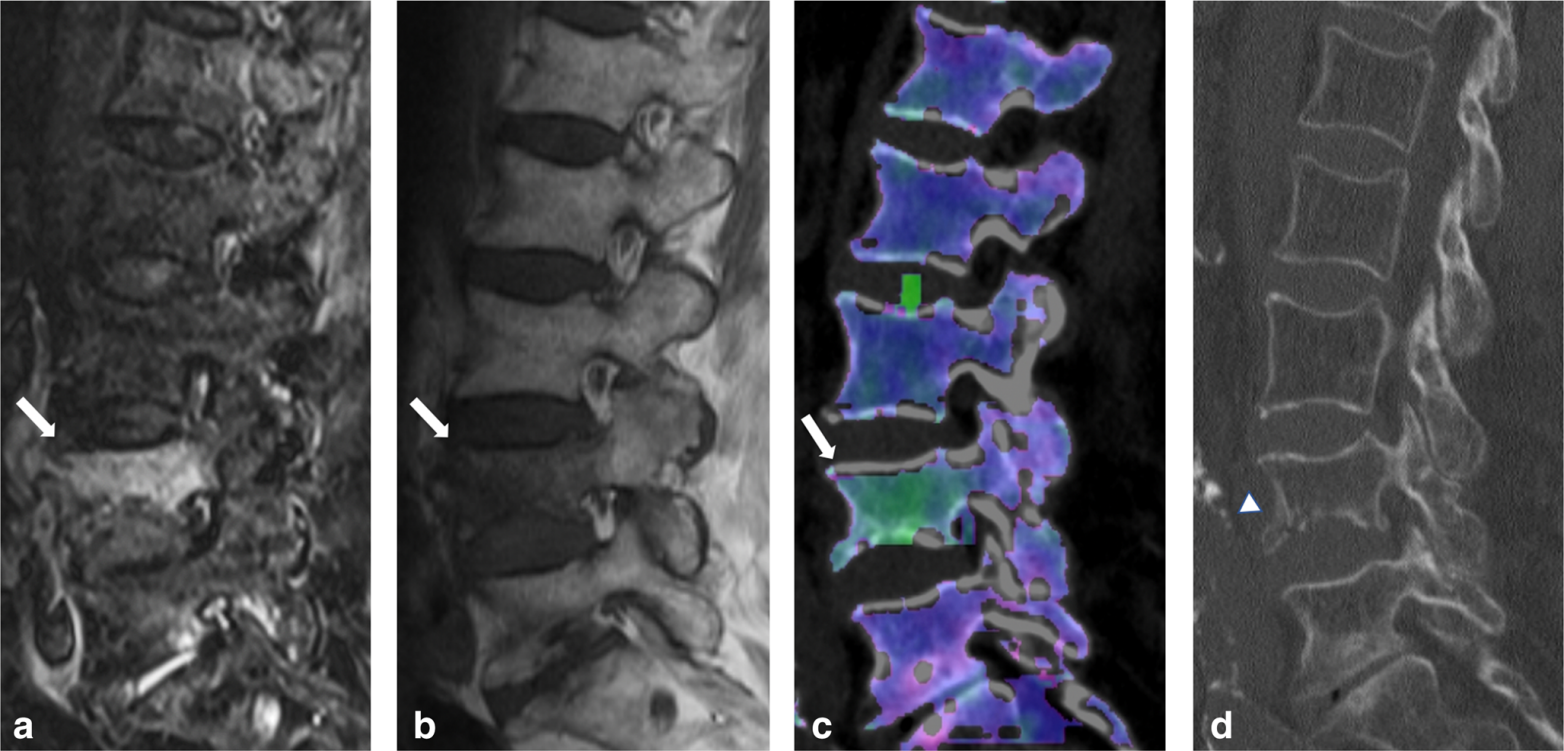
Eighty-seven-year-old woman presenting with acute spinal trauma due to a domestic fall. **a** Sagittal turbo inversion recovery magnitude (TIRM)–magnetic resonance imaging (MRI) series, **b** spin-echo (SE) T1-weighted MRI series, and **c** dual-energy computed tomography (DECT)–virtual non-calcium (VNCa) reconstructions showing bone marrow edema (BME) in all four quadrants of L4 (*arrow*). All readers were concordant in assessing BME presence (score 3 = distinct BME) and extent (score 4 = all quadrants) on both techniques. **d** In addition, sagittal conventional grayscale DECT images allowed for detection of an acute slightly dislocated fracture of the ventral ground plate of L1 (*arrowhead*) in terms of a tear drop fracture with potentially associated instability by all readers in this study. Confidence in depicting fracture lines was rated as intermediate (score 2) and high (score 3) by 5/5 readers on MR and DECT image series, respectively

MRI showed excellent overall specificity (3047/3205 [95%]), NPV (3047/3155 [97%]), and accuracy (3384/3650 [93%]), but moderate overall sensitivity (337/445 [76%]) and PPV (337/495 [68%]) for the assessment of acute traumatic fracture lines (Table 5, Fig. 3). The diagnostic confidence for fracture line identification was higher using DECT (score, 2.62) compared to MRI (score, 2.42), with a significant difference between both modalities (*p* < .001) (Table 4). Again, inter-reader agreement was good for MRI (κ = 0.71), as well as for DECT (κ = 0.73) (*p* = .12).

**Table 5:** Vertebra-based diagnostic accuracy results of every reader using magnetic resonance imaging (MRI) series for the assessment of acute vertebral fracture lines

	Sensitivity	Specificity	PPV	NPV	Accuracy
Overall	337/445 (0.76) [0.71–0.80]	3047/3205 (0.95) [0.94–0.96]	337/495 (0.68) [0.64–0.71]	3047/3155 (0.97) [0.96–0.97]	3384/3650 (0.93) [0.92–0.94]
Reader 1	70/89 (0.79) [0.69–0.87]	610/641 (0.95) [0.93–0.97]	70/101 (0.69) [0.61–0.76]	610/629 (0.97) [0.96–0.98]	680/730 (0.93) [0.91–0.95]
Reader 2	73/89 (0.82) [0.72–0.89]	607/641 (0.95) [0.93–0.96]	73/107 (0.68) [0.60–0.75]	607/623 (0.97) [0.96–0.98]	680/730 (0.93) [0.91–0.95]
Reader 3	70/89 (0.79) [0.69–0.87]	607/641 (0.95) [0.93–0.96]	70/104 (0.67) [0.59–0.74]	607/626 (0.97) [0.96–0.98]	677/730 (0.93) [0.91–0.95]
Reader 4	63/89 (0.71) [0.60–0.80]	611/641 (0.95) [0.93–0.97]	63/93 (0.68) [0.59–0.75]	611/637 (0.96) [0.94–0.97]	674/730 (0.92) [0.90–0.94]
Reader 5	61/89 (0.69) [0.58–0.78]	612/641 (0.95) [0.94–0.97]	61/90 (0.68) [0.59–0.76]	612/640 (0.96) [0.94–0.97]	673/730 (0.92) [0.90–0.94]

Statistical measures are presented as fractions and decimal values (round brackets), with 95% confidence intervals in square brackets

*PPV* positive predictive value, *NPV* negative predictive value

#### Overall acute vertebral fracture diagnosis

**Fig. 4:**
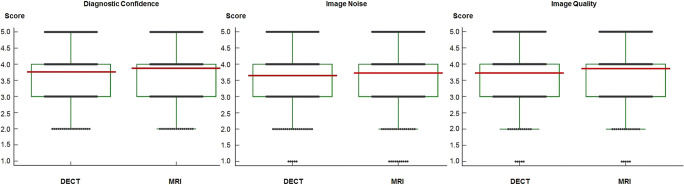
Box plots showing raters’ diagnostic confidence scores for overall diagnosis of acute vertebral fractures, image quality, and image noise using magnetic resonance imaging (MRI) and dual-energy computed tomography (DECT) image series by application of 5-point Likert scales (ranging from 1 = unacceptable to 5 = excellent). DECT image series reported similarly high scores compared to MRI without significant difference concerning overall diagnostic confidence (MRI mean score, 3.87; DECT mean score, 3.77; *p* = .15), image quality (MRI mean score, 3.85; DECT mean score, 3.74; *p* = .21), and image noise (MRI mean score, 3.73; DECT mean score, 3.66; *p* =.36). Inter-reader agreement was good for both MRI (κ = 0.72) and DECT (κ = 0.77) (*p* < .001) regarding overall diagnostic confidence, good for MRI (κ = 0.76) and excellent for DECT (κ = 0.80) (*p* < .001) concerning image quality, and good for MRI (κ = 0.73) as well as for DECT (κ = 0.73) (*p* = .35) regarding image noise

The scores regarding confidence for overall diagnosis of acute vertebral fracture, image quality, and image noise were rated with similarly high scores for MRI and DECT without significant differences and with good or excellent inter-reader agreement (Fig. 4).

### Objective image analysis

**Fig. 5:**
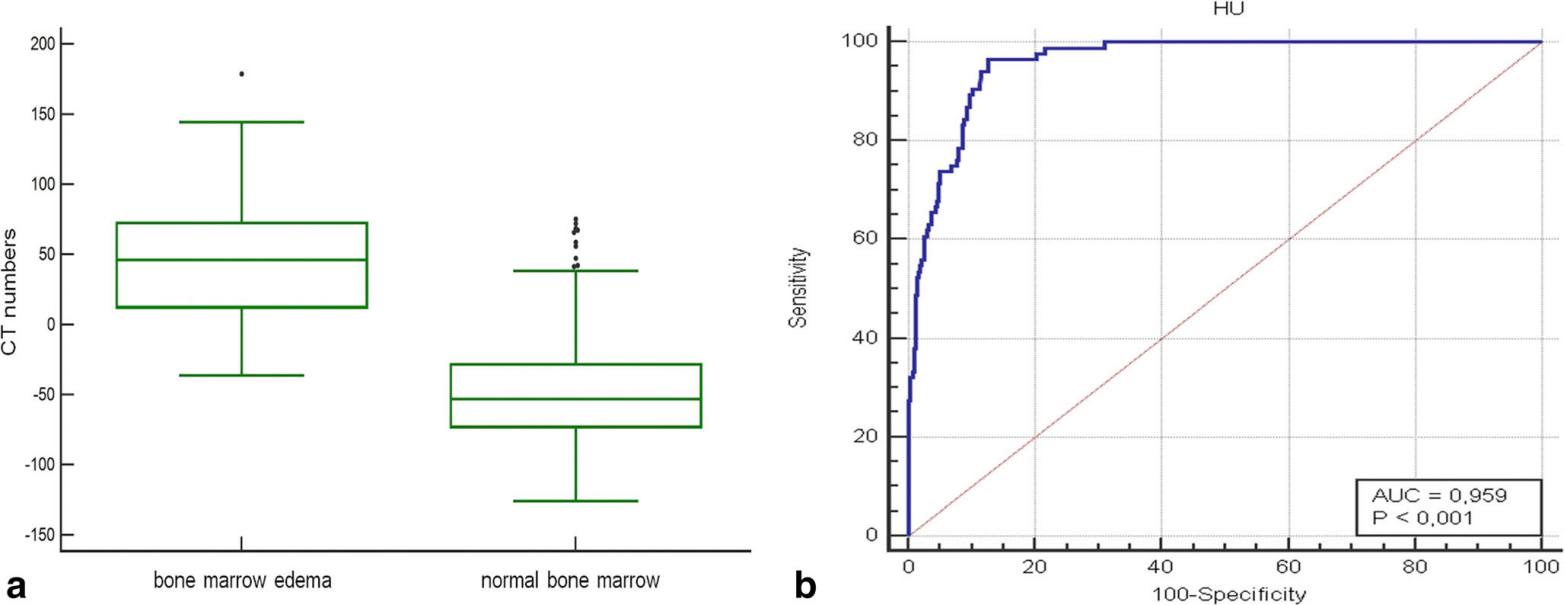
**a** Box plot shows mean computed tomography (CT) numbers on colored virtual non-calcium (VNCa) reconstructions of vertebral bodies with and without traumatic bone marrow edema (BME). CT attenuation values were significantly increased in vertebrae showing BME compared to non-edematous vertebral bodies (*p* < .001). **b** The receiver operating characteristic (ROC) curve analysis calculated from CT numbers on colored VNCa reconstructions yielded an area under the curve (AUC) of 0.96 for the differentiation of traumatic BME

Attenuation values of edematous vertebral bodies were significantly different from CT numbers of non-edematous vertebral bodies (median [IQR], 46.23 [60.60] HU and − 53.27 [44.83] HU, respectively; *p* < .001). The ROC curve analysis revealed an AUC of 0.96 and an optimal cutoff value of − 0.43 to identify vertebral BME, with an overall sensitivity of 89% (95% CI 81%, 95%) and specificity of 90% (95% CI 87%, 93%) (Fig. 5). Eventually, a significant difference between edematous and non-edematous vertebral bodies was also found when SNR values were assessed (median [IQR], 14.73 [30.90] and − 19.00 [20.95]; *p* < .01).

## Discussion

The results of our study demonstrate that DECT and MRI provide similar levels of diagnostic confidence and image quality for the assessment of acute vertebral fractures in general. DECT yielded high overall diagnostic accuracy for the depiction of BME presence and extent by application of colored VNCa series compared to MRI; both imaging approaches revealed high inter-reader agreement and equal diagnostic confidence. Regarding the identification of acute fracture lines, MRI achieved moderate sensitivity and PPV compared with DECT grayscale series; the reported readers’ diagnostic confidence was significantly higher using DECT in comparison with MRI.

Several studies demonstrated that DECT provides excellent diagnostic accuracy for depicting BME in various anatomical districts through VNCa images [18–23]. In the setting of vertebral fractures, VNCa imaging showed high levels of sensitivity, specificity, and accuracy with MRI serving as reference standard [1, 6, 8, 24, 25]. Our results are in accordance with these studies. In addition, we demonstrated high diagnostic accuracy of VNCa series for the analysis of BME extent. This analysis could be used in patient follow-up examinations, as a gradual decrease of BME has been noted both in conservatively managed and vertebroplasty-treated vertebral fractures and has been positively correlated in the former group with significant pain relief and improved quality of life [26, 27].

The objective analysis performed on VNCa series showed significantly higher CT numbers and SNR values in edematous vertebral bodies compared with non-edematous vertebral bodies. Our cutoff of − 0.43 HU to distinguish edematous from non-edematous vertebrae is different from the findings of Wang (− 80 HU), Bierry (35 HU for thoracic and 6.5 HU for lumbar vertebrae), and Petritsch (− 47 HU) [1, 8, 24, 25]. Some reasons for these discrepancies could be differences regarding the applied DECT technology, scan parameters, and post-processing algorithms, as well as the number of assessed vertebrae and patient population characteristics.

Regarding the assessment of vertebral fracture lines, MRI provided high specificity and NPV, but the sensitivity and PPV were moderate and the reported readers’ diagnostic confidence was lower compared to grayscale DECT series. Accurate morphologic depiction of fracture lines and their orientation is important for correct assessment of stability/instability as well as potentially associated disco-ligamentous injuries [28]. It is possible that the shown moderate level for ruling out fracture lines might result in potentially underestimate severity assessment of injury in certain cases where fracture lines are not visible, especially if subtle and cortical. On the other hand, MRI showed high specificity and NPV, indicating a high level at ruling in fractures in examinations with corresponding positive findings [29].

**Fig. 6:**
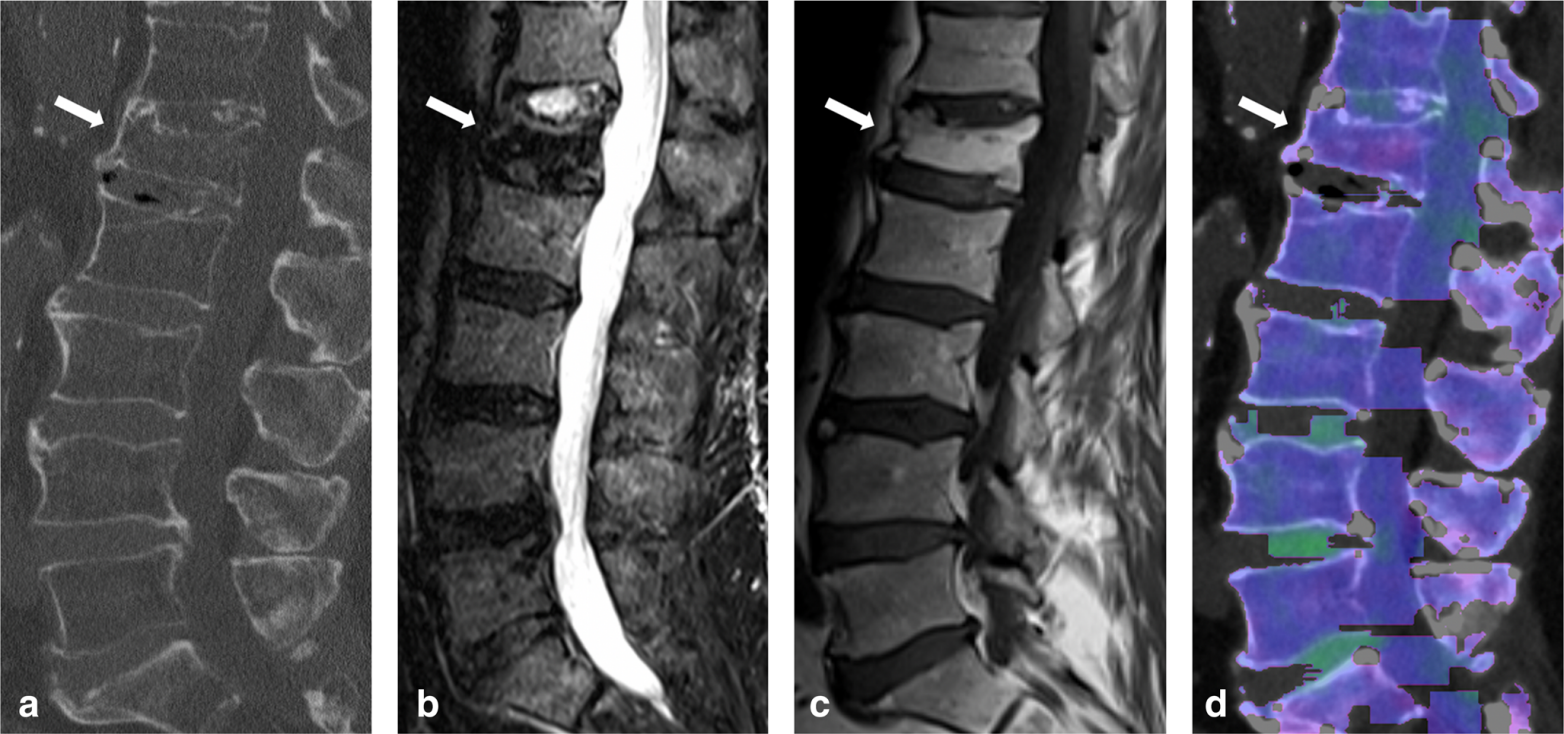
Seventy-one-year-old man presenting with spinal trauma after fall accident on a street. **a** Initially performed dual-energy computed tomography (DECT) showed a distinct compression fracture involving the superior endplate of L1 on sagittal grayscale DECT series. No further post-processing such as creation of color-coded virtual non-calcium (VNCa) reconstructions was performed in this patient in daily routine. **b**, **c** Five days later, additionally performed magnetic resonance imaging (MRI) demonstrated decreased signal intensity of L1 on sagittal turbo inversion recovery magnitude (TIRM)-MR series and an increase of signal intensity on sagittal T1-weighted spin-echo MRI (*arrows*). Therefore, the fracture was considered as old with complete fatty degeneration of bone marrow. **d** VNCa images (only reconstructed for the purpose of this study) show no evidence of bone marrow edema (BME) in L1 (*arrowhead*) (score, 0; for all readers). Furthermore, a decrease of signal intensity on VNCa images (displayed as violet area) indicates fatty degeneration of bone marrow in L1 (*arrowhead*). It is conceivable that initial reconstruction of color-coded VNCa reconstructions in daily routine would have facilitated the correct diagnosis of an old vertebral fracture

MRI currently represents the best imaging modality for a comprehensive assessment of spinal trauma, particularly considering its superior ability in evaluating neural and disco-ligamentous structures, and paravertebral soft tissues. DECT—due to the capability to visualize both fracture lines and BME—may represent an alternative to MRI when MRI is not available or its performance is not feasible, thus representing a potential “one-stop-shop examination” in specific settings of acute vertebral fractures, avoiding additional critical patient repositioning and increasing time-efficiency. This could be particularly relevant for elderly patients, who can hardly sustain long MRI examinations and often present with concurrent vertebral fracture not easily discernable from acute fractures using conventional CT (Fig. 6). Furthermore, VNCa images may provide high flexibility for additional opportunistic BME assessment in routine DECT examinations in case of incidental findings suggestive for vertebral fractures.

There are some limitations to the study. First, despite the relatively short examination interval, different findings between the two modalities concerning BME extent cannot be completely excluded. BME, albeit slowly, changes over time, also depending on the adopted therapy. Second, we did not separate thoracic and lumbar vertebrae, nor did we distinguish osteoporotic from non-osteoporotic patients. Age and type of vertebra have been considered as potential factors influencing bone marrow composition, and differences in bone mineral density could influence attenuation values on VNCa images [1, 24, 25]. All these factors could potentially influence BME evaluation and CT numbers on VNCa series. Future studies in specific population subgroups could help evaluate the real impact of these factors in clinical routine. Third, bone marrow changes could be caused by pathologies other than trauma such as malignancy or infection. However, to the best of our knowledge, other potential causes of bone marrow alteration were not present in the study population. Finally, results and conclusions are only applicable to the vendor-specific dual-source DECT technique and post-processing software, as well as to the employed imaging protocols, which are routinely used in our department in case of spinal trauma. Particularly in the assessment of fracture lines, alternative MRI protocols could have performed differently, for instance by using diverse slice thickness or sequences such as T1 spoiled gradient-echo or ultra-short echo time sequences, which have recently been shown to provide image quality comparable to CT for the evaluation of certain bone pathologies of the spine [30].

In conclusion, our study demonstrated that both DECT and MRI provide high diagnostic confidence and image quality for the assessment of acute vertebral fractures in general. DECT provided, by means of VNCa reconstructions, high diagnostic accuracy for assessing presence and extent of vertebral BME. MRI showed moderate sensitivity and lower confidence for the depiction of fracture lines. Therefore, DECT may represent a potential imaging alternative to MRI in specific settings of acute spinal trauma, especially in elderly or unstable patients and in cases of subtle or complex orientated fracture lines.

## Abbreviations

BME: Bone marrow edema
CI: Confidence interval
DECT: Dual-energy computed tomography
IQR: Interquartile range
MSK: Musculoskeletal
SD: Standard deviation
SNR: Signal-to-noise ratio
TIRM: Turbo inversion recovery magnitude
VNCa: Virtual non-calcium

## Declarations

### Guarantor

The scientific guarantor of this publication is Prof. Dr. Thomas J. Vogl (Department of Diagnostic and Interventional Radiology, University Hospital Frankfurt).

### Statistics and biometry

No complex statistical methods were necessary for this paper.

### Methodology

• retrospective

• diagnostic or prognostic study

• performed at one institution

## References

[1] Petritsch B, Kosmala A, Weng AM (2017). Vertebral compression fractures: third-generation dual-energy CT for detection of bone marrow edema at visual and quantitative analyses. Radiology.

[2] Tanigawa N, Komemushi A, Kariya S (2006). Percutaneous vertebroplasty: relationship between vertebral body bone marrow edema pattern on MR images and initial clinical response. Radiology.

[3] Han IH, Chin DK, Kuh SU (2009). Magnetic resonance imaging findings of subsequent fractures after vertebroplasty. Neurosurgery.

[4] Kazawa N (2012). T2WI MRI and MRI-MDCT correlations of the osteoporotic vertebral compressive fractures. Eur J Radiol.

[5] Booz C, Noske J, Martin SS (2019). Virtual noncalcium dual-energy CT: detection of lumbar disk herniation in comparison with standard gray-scale CT. Radiology.

[6] Kaup M, Wichmann JL, Scholtz JE (2016). Dual-energy CT-based display of bone marrow edema in osteoporotic vertebral compression fractures: impact on diagnostic accuracy of radiologists with varying levels of experience in correlation to MR imaging. Radiology.

[7] Burghardt AJ, Link TM, Majumdar S (2011). High-resolution computed tomography for clinical imaging of bone microarchitecture. Clin Orthop Relat Res.

[8] Wang CK, Tsai JM, Chuang MT, Wang MT, Huang KY, Lin RM (2013). Bone marrow edema in vertebral compression fractures: detection with dual-energy CT. Radiology.

[9] Cicero G, Ascenti G, Albrecht MH et al (2020) Extra-abdominal dual-energy CT applications: a comprehensive overview. Radiol Med. 10.1007/s11547-019-01126-5 10.1007/s11547-019-01126-5 31925704

[10] D'Angelo T, Cicero G, Mazziotti S (2019). Dual energy computed tomography virtual monoenergetic imaging: technique and clinical applications. Br J Radiol.

[11] Wortman JR, Uyeda JW, Fulwadhva UP, Sodickson AD (2018). Dual-energy CT for abdominal and pelvic trauma. Radiographics.

[12] Akisato K, Nishihara R, Okazaki H et al (2019) Dual-energy CT of material decomposition analysis for detection with bone marrow edema in patients with vertebral compression fractures. Acad Radiol. 10.1016/j.acra.2019.02.015 10.1016/j.acra.2019.02.015 30876711

[13] Pache G, Krauss B, Strohm P (2010). Dual-energy CT virtual noncalcium technique: detecting posttraumatic bone marrow lesions--feasibility study. Radiology.

[14] Frellesen C, Azadegan M, Martin SS (2018). Dual-energy computed tomography-based display of bone marrow edema in incidental vertebral compression fractures: diagnostic accuracy and characterization in oncological patients undergoing routine staging computed tomography. Invest Radiol.

[15] Liu X, Yu L, Primak AN, McCollough CH (2009). Quantitative imaging of element composition and mass fraction using dual-energy CT: three-material decomposition. Med Phys.

[16] Guggenberger R, Gnannt R, Hodler J (2012). Diagnostic performance of dual-energy CT for the detection of traumatic bone marrow lesions in the ankle: comparison with MR imaging. Radiology.

[17] Landis JR, Koch GG (1977). The measurement of observer agreement for categorical data. Biometrics.

[18] Ali IT, Wong WD, Liang T (2018). Clinical utility of dual-energy CT analysis of bone marrow edema in acute wrist fractures. AJR Am J Roentgenol.

[19] Booz C, Noske J, Albrecht MH (2019). Traumatic bone marrow edema of the calcaneus: Evaluation of color-coded virtual non-calcium dual-energy CT in a multi-reader diagnostic accuracy study. Eur J Radiol.

[20] Booz C, Noske J, Lenga L et al (2019) Color-coded virtual non-calcium dual-energy CT for the depiction of bone marrow edema in patients with acute knee trauma: a multireader diagnostic accuracy study. Eur Radiol. 10.1007/s00330-019-06304-7 10.1007/s00330-019-06304-7 31350586

[21] Chen M, Herregods N, Jaremko JL et al (2020) Bone marrow edema in sacroiliitis: detection with dual-energy CT. Eur Radiol. 10.1007/s00330-020-06670-7 10.1007/s00330-020-06670-7 32055947

[22] Rajiah P, Sundaram M, Subhas N (2019) Dual-energy CT in musculoskeletal imaging: what is the role beyond gout? AJR Am J Roentgenol. 10.2214/ajr.19.21095:1-13 10.2214/AJR.19.21095 31039024

[23] Reddy T, McLaughlin PD, Mallinson PI (2015). Detection of occult, undisplaced hip fractures with a dual-energy CT algorithm targeted to detection of bone marrow edema. Emerg Radiol.

[24] Bierry G, Venkatasamy A, Kremer S, Dosch JC, Dietemann JL (2014). Dual-energy CT in vertebral compression fractures: performance of visual and quantitative analysis for bone marrow edema demonstration with comparison to MRI. Skeletal Radiol.

[25] Wang Y, Chen Y, Zheng H, Huang X, Shan C, Bao Y (2020). Detection of different degree traumatic vertebral bone marrow oedema by virtual non-calcium technique of dual-source dual-energy CT. Clin Radiol.

[26] Piazzolla A, Solarino G, Lamartina C (2015). Vertebral bone marrow edema (VBME) in conservatively treated acute vertebral compression fractures (VCFs): evolution and clinical correlations. Spine (Phila Pa 1976).

[27] Voormolen MH, van Rooij WJ, van der Graaf Y (2006). Bone marrow edema in osteoporotic vertebral compression fractures after percutaneous vertebroplasty and relation with clinical outcome. AJNR Am J Neuroradiol.

[28] Lenchik L, Rogers LF, Delmas PD, Genant HK (2004). Diagnosis of osteoporotic vertebral fractures: importance of recognition and description by radiologists. AJR Am J Roentgenol.

[29] Mallinson PI, Coupal TM, McLaughlin PD, Nicolaou S, Munk PL, Ouellette HA (2016). Dual-energy CT for the musculoskeletal system. Radiology.

[30] Schwaiger BJ, Schneider C, Kronthaler S et al (2021) CT-like images based on T1 spoiled gradient-echo and ultra-short echo time MRI sequences for the assessment of vertebral fractures and degenerative bone changes of the spine. Eur Radiol. 10.1007/s00330-020-07597-9 10.1007/s00330-020-07597-9 PMC8213670 33443599

